# The prognostic value of serum apolipoprotein A1 levels in elderly patients with *de novo* SARS-CoV-2 omicron infection

**DOI:** 10.3389/fcimb.2025.1617266

**Published:** 2025-07-30

**Authors:** Cong Shi, Ruishuang Ma, Miao Zhou, Shujun Yang, Shengping Gong

**Affiliations:** ^1^ Stem Cell Laboratory, The First Affiliated Hospital of Ningbo University, Ningbo, Zhejiang, China; ^2^ Cancer Radiotherapy and Chemotherapy Center, The First Affiliated Hospital of Ningbo University, Ningbo, Zhejiang, China; ^3^ Department of Hematology, The First Affiliated Hospital of Ningbo University, Ningbo, Zhejiang, China

**Keywords:** omicron, SARS-CoV-2, short-term, prognosis, apolipoprotein A1

## Abstract

**Background:**

The coronavirus disease of 2019 (COVID-19) pandemic, caused by the severe acute respiratory syndrome coronavirus 2, has affected millions of people worldwide. The omicron variant is currently the predominant strain circulating worldwide. Serum apolipoprotein A1 (ApoA1) is linked to endothelial cell injury and serves as a valuable biomarker for monitoring the progression of inflammation in infected individuals. However, the potential roles of ApoA1 in the context of the omicron variant remain elusive.

**Methods:**

To investigate the prognostic value of serum ApoA1 levels at diagnosis, using mortality rate as the primary evaluation indicator, we performed a 65-day monitoring and retrospectively analyzed a cohort of 237 individuals diagnosed with omicron. Patients were categorized into two groups based on their ApoA1 levels, high and low. The Kaplan-Meier method was employed to assess overall survival (OS), while the log-rank test was utilized for comparative analysis between the groups. Additionally, both univariate and multivariate Cox proportional hazards models were applied to evaluate the prognostic significance of ApoA1 levels.

**Results:**

Our results indicated that ApoA1 levels were significantly reduced in patients infected with the omicron variant. Notably, ApoA1 levels in severe cases were lower than those in mild-to-moderate cases, with this difference reaching statistical significance. Additionally, we observed a significant increase in C-reactive protein (CRP) and beta-2 microglobulin (β2-MG) levels in individuals with decreased ApoA1 levels. Furthermore, patients with reduced ApoA1 levels exhibited a statistically significant decline in OS (*P* = 0.001). A decreased ApoA1 level (< 0.87 g/L) was identified as an independent adverse prognostic factor for OS in omicron patients, as determined by multivariate cox proportional hazards regression analysis (*P* = 0.035).

**Conclusion:**

The serum ApoA1 level at the initial diagnosis was significantly correlated with the severity and prognosis of omicron infections. Therefore, we propose that decreased levels of ApoA1 may serve as an independent negative prognostic factor in patients infected with omicron.

## Introduction

The COVID-19 pandemic, caused by the severe acute respiratory syndrome coronavirus 2 (SARS-CoV-2), has affected millions of individuals globally. This virus has the capacity to induce a range of infections, from moderate to severe. Between December 2022 and January 2023, Ningbo experienced an outbreak of the omicron BA.5 and BF.7 variants in China. Medical facilities established specialized centers for the treatment of COVID-19, specifically targeting the omicron variant of the virus. Patients received treatment in a systematic and organized manner. Several risk factors have been identified that were associated with the progression of severe COVID-19 disease, including lymphocyte subpopulations, interleukin-6 (IL-6), C-reactive protein (CRP), beta-2 microglobulin (β2-MG), and apolipoprotein E (ApoE) (Liu et al., 2021; Liu et al., 2022; Gong et al., 2023; Gong et al., 2024; Iam-Arunthai et al., 2024).

Apolipoprotein A1 (ApoA1) is a protein with a molecular weight of 45.4 kDa and serves as one of the standard indicators in lipid profiles assessed in clinical biochemistry laboratories. ApoA1 has many functions in inflammatory and immune responses. ApoA1 prevents apoptosis as well as pro-oxidative and pro-inflammatory processes in endothelial cells, promotes vasodilation, blocks platelet activation, and plays a role in innate immunity (Nazir et al., 2020). A bioinformatics study found that the more severe the disease, the lower level of ApoA1 in COVID-19 (Yu et al., 2022). The largest COVID-19 United States-based database analysis results suggested that higher ApoA1 protect against SARS-CoV-2 infection (Li et al., 2023). The prognostic significance of serum ApoA1 levels in relation to the overall survival of elderly patients diagnosed with omicron is still not well established. In light of this uncertainty, we conducted a retrospective analysis of ApoA1 levels measured at the time of diagnosis. This examination not only aims to enhance our understanding of the role of ApoA1 as prognostic marker but also seeks to contribute to the development of innovative therapeutic approaches for managing COVID-19.

## Materials and methods

### Patients

The study involved a cohort of 237 individuals diagnosed with the omicron variant of COVID-19 through virus nucleic acid test. The participants in this study had an average age of 74 years, with 150 males and 87 females. Between December 29th, 2022, and January 15th, 2023, a cohort of patients was treated at the First Affiliated Hospital of Ningbo University. This group specifically consisted of individuals who were experiencing their first episode of COVID-19 and had not undergone any prior symptomatic treatment. To monitor the progress of these patients, a follow-up period lasting 65 days was established, with a focus on mortality rates as the primary outcome measure. In order to confirm the presence of SARS-CoV-2 infection among all participants, viral nucleic acid testing was conducted, which required positive results for SARS-CoV-2 RNA. In addition to the COVID-19 patient cohort, a control group was established to provide comparative insights. This control group included peripheral blood samples from 161 healthy volunteers who were rigorously screened and confirmed to be free of any infectious diseases. The healthy individuals had no concomitant disease that interacts with serum lipid levels and hadn’t received hormone replacement treatment or use of and drugs known to impact lipid metabolism. The selection of these healthy subjects was crucial for establishing a baseline and ensuring the validity of the study outcomes. Donors were chosen from the First Affiliated Hospital of Ningbo University’s Health Management Center. In accordance with the guidelines set forth in the “Protocol for the Diagnosis and Treatment of COVID-19 Patients (Tentative 8th Edition),” the participants were classified into two groups according to the severity of their health condition: mild-to-moderate and severe (Wang et al., 2021). The main goal of the research was to assess in-hospital mortality rates. In this cohort, a total of 61 individuals passed away, whereas 176 were discharged alive from the hospital as of January 18th, 2023.

Following the guidelines established by the Helsinki Declaration, this study is a single-center retrospective analysis approved by the ethics committee at the First Affiliated Hospital of Ningbo University (2025044RS). Prior to their participation in the study, informed consent was acquired from all adult participants.

### Serum ApoA1 determination

Peripheral blood samples were obtained from subjects who had undergone a strict fasting period of no less than six hours. This ensures that the physiological state of the participants is stable, which is crucial for accurate measurements. The measurement of the serum level of ApoA1 was carried out through a turbidimetric immunoassay technique. To ensure reliability, the reagents used in the assay were evaluated and applied as per the guidelines outlined in the instructions accompanying Beckman’s ApoA1 kit. Additionally, the entire procedure was conducted using an automatic biochemical analyzer, specifically the Beckman AU5800, which enhanced precision and efficiency in the analysis.

### Absolute count of lymphocyte subsets and cytokines detection

To prevent coagulation, peripheral blood samples from each participant were treated with EDTA. The enumeration of lymphocyte subsets and cytokines was facilitated using a BD FACS Canto flow cytometer (BD, USA), which allowed for the accurate determination of absolute counts. For the identification of cytokines, the BD human Th1/Th2 cytokine kits were utilized, while the quantification of lymphocyte subsets was achieved through the application of the BD Multitest 6-color TBNK reagents. It is important to note that all experimental procedures were carried out meticulously in strict accordance with the manufacturer’s instructions, ensuring the reliability and consistency of the results obtained.

### Statistical analysis

The statistical analysis was carried out using two software programs, namely GraphPad Prism 8.0 and SPSS 26.0. The focus of the analysis was on calculating the overall survival rate of patients diagnosed with the omicron variant, starting from the time of hospital diagnosis until the cutoff date of January 18th,2023. To assess and compare overall survival rates effectively, researchers employed the Kaplan-Meier analytical technique alongside the log-rank test. This robust methodology allowed for a comparative evaluation of survival outcomes among different patient groups. In addition to the Kaplan-Meier method, a multivariable analysis was conducted using the Cox proportional hazards regression model. This regression model enabled the researchers to explore the relationship between various predictors and overall survival while controlling for other influencing factors. Two specific statistical tests were utilized in this study: the Mann-Whitney U test and Student’s t-test, which provided insights into the differences between groups in terms of categorical and continuous variables, respectively. Furthermore, chi-squared tests for categorical data, as well as the Mann-Whitney U test for continuous variables to examine the relationships effectively. The cutoff point of ApoA1 was identified using the *X*-tile program (Camp et al., 2004). The determination of the optimal threshold for differences in survival was predicated on the attainment of the most minimal *P*-value via the employment of the log-rank test, which was determined to be 0.87 g/L. In alignment with standard practices in statistical analysis, results were considered statistically significant if the *P*-value was less than 0.05, thereby providing a rigorous framework for interpreting the data collected in this study.

## Results

### Patient characteristics

The clinical data collected from a cohort of 237 individuals diagnosed with COVID-19, specifically those infected with the omicron variant, were observed over a period of 65 days. This diverse patient group consisted of 150 males and 87 females. Within this population, the median overall survival time was found to be 44 days, with a range spanning from 2 to 63 days, during which 61 fatalities were reported. In accordance with established clinical guidelines, the 237 patients were systematically categorized into two distinct groups based on the severity of their symptoms. Among them, 121 patients were identified as experiencing mild-to-moderate symptoms, while the remaining 116 were classified as suffering from severe symptoms. The mild-to-moderate group had a mean age of 73 years, comprising 70 males and 51 females. Conversely, the severe group consisted of 80 males and 36 females, with a higher median age of 78 years. Comprehensive data regarding these findings can be found in **Table 1**.

**Table 1:** Demographics and clinical characteristics of patients with omicron.

Variable	Condition on admission				Outcome			
	Mild-to-moderate patients (*n*=121)	Severe patients (*n*=116)	Statistics	*P-value*	Survivor (*n*=176)	Non-survivor (*n*=61)	Statistics	*P-value*
Male/Female, n	70/51	80/36	χ2 = 3.149	0.076	109/67	41/20	χ2 = 0.544	0.461
Age **[**years, median(quartile)**]**	73(66~84)	78(70~84)	Z=-2.105	0.035	75(67~84)	77(69.5~83.5)	Z=-1.347	0.178
Comorbidity, n								
Hypertension	72/49	73/43	χ2 = 0.293	0.588	106/70	39/22	χ2 = 0.262	0.609
Diabetes	30/91	35/81	χ2 = 0.861	0.353	46/130	19/42	χ2 = 0.572	0.450
Cardiovascular disease	10/111	3/113	χ2 = 3.683	0.055	10/166	3/58	χ2 = 0.051	0.559
Cerebrovascular disease	5/116	5/111	χ2 = 0.005	0.946	6/170	4/57	χ2 = 1.111	0.286
Pulmonary disease	3/118	3/113	χ2 = 0.003	0.638	4/172	2/59	χ2 = 0.186	0.649
Chronic kidney disease	1/120	1/115	χ2 = 0.001	0.740	2/174	0/61	χ2 = 0.699	0.551
Malignant tumor	6/115	7/109	χ2 = 0.132	0.716	9/167	4/57	χ2 = 0.182	0.745
Laboratory parameters								
WBC [×10^9^/L, median(quartile)]	6.2(4.4~9.7)	7.7(5~12.9)	Z=-2.714	0.007	6.7(4.4~10.3)	7.6(5.3~13.7)	Z=-2.017	0.044
NE [×10^9^/L, median(quartile)]	4.9(3.1~8.1)	6.2(3.8~11.2)	Z=-3.095	0.002	5.3(3.2~9)	6.5(4.2~11.7)	Z=-2.281	0.023
ALC [×10^9^/L, median(quartile)]	0.7(0.5~1.2)	0.6(0.4~0.8)	Z=-3.304	0.001	0.7(0.5~1.1)	0.5(0.3~0.7)	Z=-3.814	<0.0001
RBC [×10^9^/L, median(quartile)]	3.9(3.4~4.3)	3.9(3.4~4.4)	Z=-0.066	0.947	3.9(3.4~4.3)	3.8(3.2~4.5)	Z=-0.452	0.651
HB [g/L, median(quartile)]	121(107~131)	120(106~139)	Z=-0.342	0.732	121(108~133)	119(99.5~141)	Z=-0.158	0.874
PLT [×10^9^/L, median(quartile)]	182(137~242)	192(140~253)	Z=-0.603	0.547	191(143~249)	155(121~240)	Z=-2.050	0.040
CRP [mg/L, median(quartile)]	31.6(12.9~61.5)	55.8(28.3~113)	Z=-4.131	<0.0001	38.6(16.4~69.8)	63.7(24.6~116.5)	Z=-3.000	0.003
β2-MG [mg/L, median(quartile)]	3.1(2.2~4.4)	4.5(2.6~7.3)	Z=-3.649	<0.0001	3.1(2.2~4.6)	5.9(3.1~12)	Z=-5.574	<0.0001
ApoE [mg/L, median(quartile)]	45.9(38.1~57.8)	52.3(42~67.5)	Z=-2.713	0.007	46.8(38.7~59.5)	54.1(44~75.2)	Z=-2.873	0.004
ApoA1 [g/L, median(quartile)]	0.9(0.8~1.1)	0.8(0.7~0.9)	Z=-4.348	<0.0001	0.9(0.7~1)	0.8(0.6~0.9)	Z=-3.876	<0.0001
Cytokines								
IL-6 [pg/ml, median(quartile)]	14.4(9.6~29.8)	36.9(15~94.2)	Z=-4.578	<0.0001	17.9(10.1~52.5)	42.4(18.6~132.5)	Z=-4.250	<0.0001
IL-10 [pg/ml, median(quartile)]	7.9(5.4~12.9)	13(8.5~24.3)	Z=-4.739	<0.0001	9.5(6.5~15.4)	14.2(6.9~44.7)	Z=-3.034	0.002
Lymphocyte subpopulation								
CD3 absolute value [/μl, median(quartile)]	322(207~627)	207(152~375)	Z=-3.073	0.002	297(187~575)	198(141~324)	Z=-2.447	0.014
CD4 absolute value [/μl, median(quartile)]	176(108~321)	112(77.5~219)	Z=-2.559	0.011	154(95~296)	115(77~229)	Z=-1.280	0.201
CD8 absolute value [/μl, median(quartile)]	126(68~247)	70(36.5~147)	Z=-2.825	0.005	119(59~235)	68(31~111)	Z=-3.061	0.002
NK absolute value [/μl, median(quartile)]	130(73~209)	90(46~151)	Z=-2.418	0.016	111(71~208)	83(37~151)	Z=-2.475	0.013
CD19 absolute value [/μl, median(quartile)]	73(33~131)	64(37.5~108.5)	Z=-0.400	0.689	72(36~127)	70(35~101)	Z=-0.430	0.667

WBC, white blood count; NE, neutrophil; ALC, absolute lymphocyte count; RBC, red blood count; HB, hemoglobin; CRP, C reactive protein; β2-MG, beta-2 microglobulin; ApoE, apolipoprotein E; ApoA1, apolipoprotein A1; IL-6, interleukin-6; IL-10, interleukin-10.

### Serum ApoA1 level in peripheral blood of omicron patients

**Figure 1:**
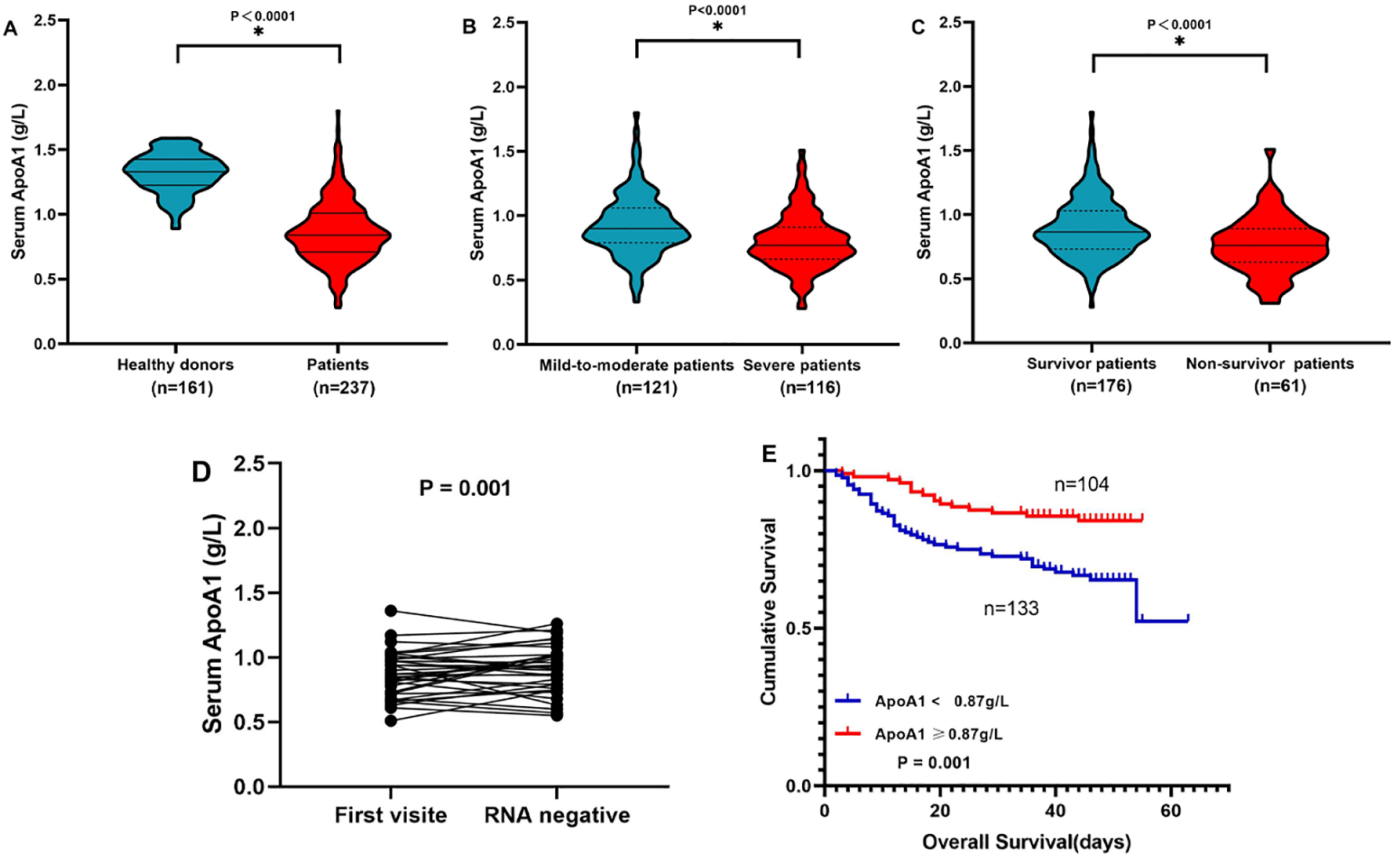
**(A)** Compare serum ApoA1 between 161 healthy donors and 237 patients. **(B)** Compare serum ApoA1 between 121 mild-to-moderate patients and 116 severe patients. **(C)** Compare serum ApoA1 between 176 survivor patients and 61 non-survivor patients. **(D)** The dynamic changes of serum ApoA1 in omicron patients whose viral nucleic acid test turned negative. **(E)** Overall survival according to serum ApoA1 in omicron patients. * P<0.05.

In our study cohort, individuals infected with the omicron variant of COVID-19 exhibited markedly reduced serum levels of ApoA1 compared to those who were not infected. Specifically, the median ApoA1 concentration among patients identified with the omicron variant was significantly lower at 0.84 g/L, compared to 1.33 g/L observed in 161 healthy donors, with a statistical significance indicated by *P* < 0.0001 (**Figure 1A**). Furthermore, when analyzing patient conditions based on severity, we found that patients experiencing severe manifestations of the disease had considerably lower serum ApoA1 levels than those classified as mild-to-moderate cases. Notably, a stark contrast was observed in serum ApoA1 levels between patients who did not survive and those who did specifically, the ApoA1 levels among non-survivors showed a significant decrease relative to survivors, with this difference achieving a statistical significance of *P* < 0.0001 (**Figures 1B, C**).

### The relationship between serum ApoA1 level and other factors in clinics and laboratory

The SARS-CoV-2 virus has recently given rise to a variant known as the omicron variant. A study aimed at investigating the potential associations between the levels of ApoA1 and various laboratory and clinical characteristics in patients categorized the subjects into two distinct groups. The findings reveal that the group with diminished ApoA1 levels demonstrated significantly elevated levels of CRP, with a *P*-value of 0.001, indicating a robust correlation between lower ApoA1 and increased inflammatory markers. Furthermore, this group exhibited notably higher levels of β2-MG with the same level of statistical significance (*P* = 0.001) and presented a significantly lower absolute lymphocyte count (ALC), marked by a *P*-value of 0.008. Additionally, patients with reduced ApoA1 levels had lower counts in several other key hematological measurements, including a diminished red blood cell count (RBC) with a *P*-value of 0.003 and a lower hemoglobin (HB) level, which had a *P*-value of 0.015. Moreover, the absolute count of CD3 lymphocytes was significantly reduced (*P* = 0.001), as was the absolute count of CD4 lymphocytes (*P* = 0.016). The study also found a stark decrease in the absolute count of CD8 lymphocytes, with a highly significant *P*-value of less than 0.0001, as well as a decrease in the absolute count of CD19 lymphocytes (*P* = 0.006). In contrast, as reflected in **Table 2**, other examined variables did not show significant differences between the two analyzed groups, highlighting specific relationships with ApoA1 levels.

**Table 2:** Comparison between omicron with low ApoA1 group and high ApoA1 group in 237 patients.

Variable	Low ApoA1 group (*n*=133)	High ApoA1 group (n=104)	Statistics	*P-value*
Male/Female, n	95/38	55/49	χ2 = 8.638	0.003
Age **[**years, median (quartile)**]**	78 (70~85)	73 (66~82)	Z=-2.422	0.015
Comorbidity, n				
Hypertension	86/47	59/45	χ2 = 1.546	0.214
Diabetes	32/101	33/71	χ2 = 1.725	0.189
Cardiovascular disease	6/127	7/97	χ2 = 0.555	0.456
Cerebrovascular disease	5/128	5/99	χ2 = 0.159	0.690
Pulmonary disease	4/129	2/102	χ2 = 0.278	0.698
Chronic kidney disease	0/133	2/102	χ2 = 2.579	0.192
Malignant tumor	9/124	4/100	χ2 = 0.960	0.398
Laboratory parameters				
WBC [×10^9^/L, median (quartile)]	7.7 (5.0~11.4)	6.2 (4.4~10.2)	Z=-1.630	0.103
NE [×10^9^/L, median (quartile)]	6.4 (3.8~9.8)	4.9 (3.2~9.0)	Z=-1.797	0.072
ALC [×10^9^/L, median (quartile)]	0.6 (0.4~0.8)	0.7 (0.5~1.1)	Z=-2.641	0.008
RBC [×10^9^/L, median (quartile)]	3.8 (3.2~4.3)	4.1 (3.6~4.4)	Z=-2.950	0.003
HB [g/L, median (quartile)]	117 (104~134)	124 (112~137)	Z=-2.441	0.015
PLT [×10^9^/L, median (quartile)]	180 (130~248)	194 (142~251)	Z=-1.144	0.253
CRP [mg/L, median (quartile)]	52.2 (24.1~109.9)	31.2 (11.2~62)	Z=-3.477	0.001
β2-MG [mg/L, median (quartile)]	3.9 (2.6~6.7)	3 (2.2~4.9)	Z=-3.313	0.001
ApoE [mg/L, median (quartile)]	48.2 (39.1~60.3)	49 (39.7~63.1)	Z=-1.059	0.290
ApoA1 [g/L, median (quartile)]	0.7 (0.6~0.8)	1 (0.9~1.2)	Z=-13.206	<0.0001
Cytokines				
IL-6 [pg/ml, median (quartile)]	23.4 (12.8~67.5)	18.2 (10~57.8)	Z=-1.725	0.085
IL-10 [pg/ml, median (quartile)]	10.6 (7~20.9)	9.8 (6.2~15.5)	Z=-1.788	0.074
Lymphocyte subpopulation				
CD3 absolute value [/μl, median (quartile)]	207 (152~374)	350 (220~627)	Z=-3.426	0.001
CD4 absolute value [/μl, median (quartile)]	117 (79~216)	187 (103~335)	Z=-2.419	0.016
CD8 absolute value [/μl, median (quartile)]	75 (32~136)	147 (70~274)	Z=-3.906	<0.0001
NK absolute value [/μl, median (quartile)]	97 (59~177)	114 (68~209)	Z=-1.409	0.159
CD19 absolute value [/μl, median (quartile)]	61 (30~96)	91 (49~132)	Z=-2.744	0.006

WBC, white blood count; NE, neutrophil; ALC, absolute lymphocyte count; RBC, red blood count; HB, hemoglobin; CRP, C reactive protein; β2-MG, beta-2 microglobulin; ApoE, apolipoprotein E; ApoA1, apolipoprotein A1; IL-6, interleukin-6; IL-10, interleukin-10.

### Changes in the levels of ApoA1 in omicron patients during follow-up

A study was conducted involving thirty-five patients recently diagnosed with the omicron variant of the virus. These patients underwent a two-week monitoring period, during which their health and biomarker levels were closely observed. Upon completion of this observation period, a follow-up assessment revealed that the majority of patients who tested negative for the virus through nucleic acid testing exhibited a significant increase in their ApoA1 levels. This improvement is visually represented in **Figure 1D**, illustrating the positive correlation between viral clearance and ApoA1 levels in the patients.

### Low ApoA1 level was associated with a poor prognosis

Patients with diminished levels of ApoA1 experienced a significantly shorter median overall survival compared to those with elevated ApoA1 levels, with survival times of 43 days and 45.5 days, respectively. This difference was statistically significant, with a *P*-value of 0.001, as illustrated in **Figure 1E**.

This finding suggests that ApoA1 may play a crucial role in the prognosis of these patients, with lower levels potentially indicating a more severe disease state or poorer clinical outcomes. Furthermore, the univariate analysis revealed a robust and statistically significant inverse relationship between overall survival and various clinical variables. Specifically, increased concentrations of CRP, IL-6, and IL-10 were associated with poorer survival outcomes, all exhibiting *P*-values below 0.0001. Additionally, patients with elevated WBC counts, higher NE counts, and reduced ALC, HB, and PLT counts also demonstrated worse survival. The data indicated that elevated β2-MG and ApoE levels, alongside decreased ApoA1 levels, correlated with adverse survival. Notably, diminished absolute counts of CD3 and CD8 lymphocytes, as well as lower counts of NK lymphocytes, also reflected significant associations with survival. The factors in the univariate analyses with *P* value less than 0.1 were absorbed into the multivariate analyses. Aside from the aforementioned parameters, lower CD4 lymphocyte absolute counts (*P* = 0.052), lower CD19 lymphocyte absolute counts (*P* = 0.053) and lower RBC counts (*P* = 0.06) were also included in the multivariate analyses. In the context of multivariate analyses, the findings highlighted that independent adverse prognostic factors contributing to diminished overall survival included lower absolute counts of NK lymphocytes (*P* = 0.04), higher levels of β2-MG (*P* < 0.0001), and decreased levels of ApoA1 (*P* = 0.035). These results, presented in **Table 3**, confirm that these specific biomarkers can serve as pivotal indicators of survival, suggesting that targeting these elements may enhance therapeutic strategies to improve patient outcomes in this population.

**Table 3:** Univariate and multivariate analyses of different prognostic parameters for overall survival of 237 patients with omicron.

Variables	Univariate analysis for OS		Multivariate analysis for OS		
	*P-value*	95%CI	*P-value*	*HR*	95%CI
Age ≥ 60(years)	0.299	47.534-53.382	–	–	–
Gender (male)	0.461	47.151-53.372	–	–	–
CRP ≥ 83.24mg/L	<0.0001	37.539-48.777	0.802	1.105	0.506-2.410
IL-6 ≥ 15.45pg/ml	<0.0001	51.506-58.051	0.457	1.485	0.524-4.209
IL-10 ≥ 11.9pg/ml	<0.0001	44.131-55.451	0.328	1.586	0.630-3.996
CD3 absolute value < 236/μl	0.001	34.445-43.557	0.764	1.226	0.325-4.632
CD4 absolute value < 106/μl	0.052	35.794-45.702	0.759	1.156	0.458-2.921
CD8 absolute value < 115/μl	<0.0001	34.822-43.133	0.088	3.196	0.840-12.170
NK absolute value < 77/μl	0.006	34.416-44.034	0.040	2.219	1.036-4.753
CD19 absolute value < 118/μl	0.053	38.696-45.297	0.819	1.170	0.306-4.471
WBC ≥ 14.9×10^9^/L	<0.0001	27.163-43.098	0.270	3.833	0.352-41.676
NE ≥ 3.75×10^9^/L	0.001	26.675-43.257	0.930	1.115	0.097-12.877
ALC < 0.7×10^9^/L	0.002	38.118-45.041	0.862	1.107	0.354-3.462
RBC < 4.13×10^9^/L	0.060	37.794-46.293	0.817	1.121	0.426-2.955
HB < 133.5g/L	0.024	33.763-46.444	0.898	1.081	0.329-3.552
PLT < 152.5×10^9^/L	0.011	35.264-45.515	0.479	1.359	0.581-3.176
Hypertension	0.521	46.918-53.874	–	–	–
Cardiopathy	0.846	33.954-54.969	–	–	–
Diabetes	0.422	39.742-53.012	–	–	–
β2-MG ≥ 4.72mg/L	<0.0001	32.425-43.408	<0.0001	4.222	1.878-9.488
ApoE > 49.8mg/L	0.001	37.945-45.183	0.193	1.750	0.754-4.061
ApoA1 < 0.87g/L	0.001	41.787-50.424	0.035	2.776	1.073-7.183

WBC, white blood count; NE, neutrophil; ALC, absolute lymphocyte count; RBC, red blood count; HB, hemoglobin; CRP, C reactive protein; β2-MG, beta-2 microglobulin; ApoE, apolipoprotein E; ApoA1, apolipoprotein A1; IL-6, interleukin-6; IL-10, interleukin-10.

## Discussion

SARS-CoV-2 is highly contagious, and COVID-19 poses a significant risk to life and health globally. Changes in blood lipid levels may serve as significant and accessible biomarkers for assessing the severity of COVID-19 (Ochoa-Ramírez et al., 2024). The presence and concentration of specific lipids in the blood can provide valuable insights into the body’s inflammatory response and overall health status during infection. Monitoring these lipid levels may deepen our understanding of the disease’s progression and could potentially assist in identifying patients at higher risk for severe outcomes. It is crucial to halt the spread of the epidemic promptly, accurately assess the prognosis of patients, and deliver more effective therapies for those with poor prognoses. This study’s findings indicate that patients diagnosed with omicron have lower serum ApoA1 levels compared to those classified as healthy.

Our research revealed that individuals experiencing severe symptoms exhibited reduced serum ApoA1 levels prior to therapy, in contrast to those with mild-to-moderate symptoms. Additionally, serum ApoA1 levels were significantly lower in patients who did not survive compared to those who did. Furthermore, decreased serum ApoA1 levels correlated with increased levels of CRP, ApoE, and β2-MG, as well as decreased absolute counts of ALC, CD3 lymphocytes, CD4 lymphocytes, CD8 lymphocytes, and CD19 lymphocytes. Notably, serum ApoA1 levels were significantly elevated in thirty-five newly diagnosed patients. However, following two weeks of treatment, these levels decreased markedly, coinciding with a negative result on the virus nucleic acid test. Our analysis of ApoA1 levels in patients treated at our hospital indicates that fluctuations in ApoA1 may serve as prognostic indicators. We found a positive correlation between reduced serum ApoA1 levels and shorter survival times in individuals infected with the omicron variant. This suggests that lower serum ApoA1 concentrations could signify a poor prognosis for those diagnosed with omicron. Results from cox regression analysis demonstrate that diminished ApoA1 levels are a unique and statistically significant prognostic marker for patients affected by omicron.

ApoA1 is primarily produced in two main organs: the liver and the small intestine. This protein plays a critical role in lipid metabolism and serves as the chief component of high-density lipoprotein (HDL) found in plasma (Michalaki et al., 2005). Lower HDL and ApoA1 levers are associated with higher severity and mortality rates (Rezaei et al., 2022). HDL is a dynamic and adaptable lipoprotein particle that aids the innate immune system in combating early-life acute events such as infection and injury. HDL has antiviral function, preventing viruses from entering or merging with host cells, therefore inhibiting their reproduction cycle during viral infections similar to COVID-19 (Rani et al., 2024). ApoA1 is essential for various physiological processes, including the transport of cholesterol from tissues to the liver for excretion, thereby contributing to overall cardiovascular health. In addition to its role in regulating cholesterol and protecting against cardiovascular disease, ApoA1 also plays a significant role in inflammatory and immune responses (Wang et al., 2016; Nazir et al., 2020). ApoA1 has been shown to inhibit lipid peroxidation and reduce the secretion of inflammatory factors, thereby exhibiting significant anti-inflammatory effects. As an acute-phase response protein, its levels increase rapidly and markedly, serving as a crucial biomarker for tissue inflammation and injury. Shen et al (Shen et al., 2020). found that ApoA1 was down-regulated in COVID-19 patients, and had a negative correlation with CRP. Additionally, ApoA1 plays a pivotal role in various biological processes by inducing post-translational modifications of apolipoproteins and functions to counteract the cascade of inflammatory factors (Liu et al., 2018). Previous study has been reported that ApoA1 rapidly disrupts membrane lipid rafts, and dampens the PI3K/Akt signaling pathway, thereby suppressing the progression of inflammation (lqbal et al., 2016).

Researches had indicated that ApoA1 levels can serve as a predictor of COVID-19 severity, with adequate levels potentially acting as a protective factor against severe outcomes (Ulloque-Badaracco et al., 2021; Zhu et al., 2021). Our findings supported this assertion, revealing that severe cases and non-survivors exhibited significantly lower ApoA1 levels compared to mild to moderate cases and survivors. Additionally, our study demonstrated that lower ApoA1 levels in patients infected with the omicron variant was correlated with an increased risk of mortality and reduced overall survival rates. Consequently, the serum level of ApoA1 might serve as a predictive indicator for the severe stages of coronavirus infection, thereby informing patient prognosis. This underscored the necessity for screening and monitoring to facilitate appropriate interventions.

The current study acknowledges several limitations that must be addressed. First, the clinical data used for analysis were somewhat restricted, which may impact the robustness of the findings. Due to the population was elderly individuals, the data from this study may be influenced by demographic-based. Additionally, as a retrospective study, it may lead to bias in participant selection and the presence of confounding variables. Moreover, it is important to note that the research did not explore the underlying mechanisms connecting ApoA1 with the severity and prognosis of omicron COVID-19, leaving a gap in understanding the biological processes involved. Finally, the findings would benefit significantly from replication in larger cohort studies, as this investigation was exploratory in nature and relied on a relatively small sample size. Expanding the scope of the research could provide more definitive insights and enhance the validity of the results.

## Data Availability

The raw data supporting the conclusions of this article will be made available by the authors, without undue reservation.
